# The predictive value of aGAPSS score combined with clinical factors for thrombosis in systemic lupus erythematosus

**DOI:** 10.3389/fcimb.2026.1909515

**Published:** 2026-09-18

**Authors:** Juan Shi, Libo Ouyang, Qiong Ma, Peiming Zheng

**Affiliations:** 1 Department of Laboratory Medicine, Henan Provincial People’s Hospital, Zhengzhou, Henan, China; 2 Department of Blood Transfusion, Zhumadian Central Hospital, Zhumadian, Henan, China

**Keywords:** antiphospholipid antibodies, antiphospholipid syndrome score, risk prediction, systemic lupus erythematosus, thrombosis

## Abstract

**Purpose:**

This study evaluated the predictive value of antiphospholipid antibodies (aPLs) combined with the adjusted global antiphospholipid syndrome score (aGAPSS) for thrombosis in hospitalized systemic lupus erythematosus (SLE) patients.

**Methods:**

A retrospective analysis included 309 SLE patients (65 with in-hospital thrombosis, 244 without). Demographic, clinical, and serological data were collected. Least absolute shrinkage and selection operator (LASSO) regression and multivariate logistic regression identified independent risk factors. A combined predictive model was constructed and internally validated using bootstrap.

**Results:**

Patients with thrombosis were older, had higher smoking prevalence, LA ratio, and aGAPSS (all *P* < 0.05). Disease activity was significantly higher in the thrombosis group, as reflected by a higher mean SLEDAI score (14.29 ± 2.88 vs. 12.45 ± 1.62, *P* < 0.001). Multivariate analysis identified age (OR = 1.053), smoking (OR = 2.945), SLEDAI (OR = 1.312), LA ratio (OR = 17.432), and aGAPSS (OR = 1.514) as independent predictors. The combined model (aGAPSS + clinical covariates) achieved an area under the curve (AUC) of 0.832, significantly outperforming aGAPSS alone (AUC = 0.693; ΔAUC = 0.139, *P* < 0.001), with NRI of 0.356 and IDI of 0.142.

**Conclusion:**

Integrating aGAPSS with clinical factors provides good discriminative ability for in-hospital thrombosis risk stratification in hospitalized SLE patients, offering a practical tool that may inform surveillance intensity and prophylactic consideration.

## 1 Introduction

Systemic lupus erythematosus (SLE) is a chronic multi-system autoimmune disease characterized by the production of autoantibodies, deposition of immune complexes, and a wide range of clinical manifestations from rashes and arthritis to severe kidney, neurological, and cardiovascular involvement (Nossent et al., 2024; Yang et al., 2024). Although treatments such as antimalarial drugs, glucocorticoids, and biologics are available, current therapeutic strategies remain limited in preventing thrombotic complications, which are a major cause of morbidity and mortality in SLE patients (Feng et al., 2024; Cantera Estefanía et al., 2025). A significant contributor to this therapeutic shortcoming is the difficulty in accurately identifying the subset of patients at highest imminent risk for thrombosis, which hampers targeted and timely prophylactic interventions. Therefore, improving risk prediction models is a critical step toward mitigating the thrombotic burden in SLE. Despite advances in management strategies, accurately predicting which patients will experience thrombotic events remains a clinical challenge (Betensky et al., 2024). Current risk assessments often rely on the presence of antiphospholipid antibodies (aPLs), but their predictive value alone is not ideal (Yelnik et al., 2025).

The pathogenesis of thrombosis in SLE is multifactorial, involving complex interactions between immune dysregulation, inflammation, and endothelial dysfunction (Hisada and Atsumi, 2023; Sciascia et al., 2023). A key mechanism involves antiphospholipid syndrome (APS), often secondary to SLE (Akyüz Dağlı et al., 2024). It is well established that the presence of aPLs, such as anticardiolipin antibodies (aCL), anti-β2-glycoprotein I antibodies (a-β2GPI), and lupus anticoagulant (LA), promotes a prothrombotic state by activating endothelial cells, platelets, and complement (Mihailovic et al., 2023; Murray et al., 2024). In addition to aPLs, traditional cardiovascular risk factors and overall SLE disease activity are also associated with accelerating the atherosclerotic process and increasing the risk of thrombosis (Venturelli et al., 2024). The adjusted global antiphospholipid syndrome score (aGAPSS) was developed to integrate these aPL characteristics with traditional risk factors into a single, quantifiable score, providing a promising tool for more comprehensive risk assessment (Gao et al., 2024). While individual risk factors have been examined, few studies have systematically combined clinical covariates with the established aGAPSS system to construct robust predictive models for SLE-related thrombosis. The rationale for this integrated approach is that the aGAPSS provides a framework of both serological (binary aPL positivity) and traditional cardiovascular risk factors, whereas clinical covariates such as disease activity capture additional dimensions of thrombotic risk not fully reflected in the score. Combining these complementary dimensions of risk—the established aGAPSS and clinical factors—may yield a more powerful and clinically useful predictive tool than either component alone.

Therefore, this study aims to evaluate the incremental predictive value of adding the clinical covariates to the aGAPSS for in-hospital thrombosis risk stratification in SLE patients. We further utilized multivariate logistic regression to construct a combined predictive model. The innovation of this study lies in quantifying the additive value of clinical factors to a validated serological risk score, providing practical tools for in-hospital​ thrombosis risk stratification in hospitalized SLE patients, which may inform clinical decisions regarding thromboprophylaxis.

## 2 Materials and methods

### 2.1 Study design

A retrospective analysis was conducted on 309 SLE patients who were hospitalized in our hospital from January 2020 to December 2023. All included patients were adults (age ≥ 18 years) and met the SLE diagnostic criteria of the 2012 Systemic Lupus International Collaborating Clinics (SLICC) classification criteria (Petri et al., 2012), with complete medical records and no missing data. Patients with other systemic autoimmune diseases, with prior thrombosis history, and without testing for aPLs were excluded.

This study strictly adhered to the Declaration of Helsinki and relevant ethical guidelines for medical research. The clinical data of all included patients were anonymized and used solely for academic research purposes, ensuring patient privacy protection. The research protocol was reviewed and approved by the Medical Ethics Committee of Henan Provincial People’s Hospital (Approval Number: 2024-1-112). As the study was designed retrospectively and did not involve any additional invasive procedures, the ethics committee approved the waiver of informed consent from the patients.

### 2.2 Data collection

Demographic and disease characteristics, routine coagulation indicators, aPLs positivity rates, and aGAPSS of SLE patients at admission were collected through the hospital’s electronic medical record system. Additionally, new thromboembolic events during the patients’ hospital stay were also recorded. Prothrombin time (PT), activated partial thromboplastin time (APTT), thrombin time (TT), and fibrinogen (FIB) levels were measured using an automated coagulation analyzer (ACL TOP 750, Werfen, USA).

aPLs testing included aCL IgG and IgM, anti-β2-glycoprotein I (a-β2GPI) antibody IgG and IgM, and lupus anticoagulant (LA). ACA and a-β2GP1 antibody tests were performed using the EUROIMMUN Anti-Cardiolipin and Anti-β2-Glycoprotein I ELISA Kits (E0356-9601, E0357-9601). The positive threshold for aCL IgG/IgM was ≥40 phospholipid units (GPLU/MPLU), respectively, and for β2GPI IgG/IgM, it was ≥20 units (GBU/MBU), respectively.LA testing was performed using the Siemens Lupus Anticoagulant Detection Kit (00074821). The dilute Russell’s viper venom time (dRVVT) assay was used for both the initial lupus anticoagulant screening test (LA1) and the confirmatory lupus anticoagulant test (LA2). A ratio of LA1/LA2 > 1.2 was defined as positive.

The aGAPSS calculated risk based on five criteria: hyperlipidemia was assigned 3 points, hypertension 1 point, positive aCL IgG and IgM antibodies 5 points, positive a-β2GPI antibody IgG and IgM as well as LA positivity each receive 4 points. The maximum score was 17 points, with a score of 0 assigned if none of these conditions were present (Sciascia et al., 2013).

### 2.3 Patient grouping

Based on the presence or absence of new arterial or venous thromboembolic events during hospitalization, 309 SLE patients were defined into a non-thrombosis group (n=244) and a thrombosis group (n=65). The clinical diagnosis of thromboembolic events was made by the treating physicians based on compatible symptoms and objective imaging evidence (Doppler ultrasound, computed tomography angiography, magnetic resonance angiography, or pulmonary ventilation/perfusion scintigraphy). The types of events included—macrovascular arterial thrombosis (e.g., acute myocardial infarction, ischemic stroke, peripheral arterial occlusion) and venous thromboembolism (e.g., deep vein thrombosis, pulmonary embolism)—were selected with reference to the clinical event definitions used in the 2023 ACR/EULAR APS classification criteria (Barbhaiya et al., 2023), to ensure alignment with standardized research terminology. However, we did not apply the full weighted classification algorithm of the 2023 criteria to define individual events, as these criteria are intended for overall APS classification rather than for bedside diagnosis of acute thrombosis. The most commonly encountered manifestations in our cohort included angina pectoris, acute myocardial infarction, cerebrovascular accident, transient ischemic attack, digital gangrene, retinal artery occlusion, intermittent claudication (arterial), as well as lower extremity deep vein thrombosis, pulmonary embolism, retinal vein occlusion, and Budd-Chiari syndrome (venous). Less common arterial events (e.g., splenic or renal artery thrombosis) were not observed in the present cohort.

### 2.4 Statistical analysis

All statistical analyses were performed using R software (Version 4.3.1; R Foundation for Statistical Computing, Vienna, Austria). Continuous variables were tested for normality using the Shapiro–Wilk test and presented as mean ± standard deviation (M ± SD); comparisons between groups were conducted using independent samples t-tests. Categorical variables were expressed as frequencies and percentages [n(%)] and compared using Pearson’s chi-square tests. A two-tailed α=0.05 was considered statistically significant.

To select predictors for the multivariate analysis, least absolute shrinkage and selection operator (LASSO) regression was applied to all candidate variables using 10-fold cross-validation; the optimal penalty parameter λ was chosen as the largest value within one standard error of the minimum binomial deviance (λ.1se) to balance model fit and parsimony. Variables with non-zero coefficients were then entered into multivariate logistic regression to identify independent risk factors for in-hospital thrombosis, with results reported as odds ratios (OR) and 95% confidence intervals (CI).

Model discrimination was assessed by area under the curve (AUC), and comparisons between nested models were performed using DeLong’s test for correlated receiver operating characteristic (ROC) curves. To evaluate incremental predictive value, the net reclassification improvement (NRI) and integrated discrimination improvement (IDI) were calculated using the PredictABEL package. For internal validation, bootstrap resampling with 1,000 iterations was performed to derive optimism-corrected performance metrics. Clinical utility was assessed via decision curve analysis by quantifying net benefit across a range of threshold probabilities.

## 3 Results

### 3.1 General information

The age in the thrombosis group was higher than that in the non-thrombosis group (P = 0.009) (**Table 1**). There were no significant differences in gender distribution, BMI, and history of alcohol consumption between the two groups (gender: P = 0.130; BMI: P = 0.119; alcohol consumption: P = 0.154). The history of smoking was significantly higher in the thrombosis group compared to the non-thrombosis group (P = 0.002), indicating a higher proportion of patients with a smoking history in the thrombosis group. In terms of cardiovascular risk factors, arterial hypertension was significantly higher in the thrombosis group compared to the non-thrombosis group (P = 0.007). There was no statistically significant difference in hyperlipidemia between the two groups (P = 0.194), and there were no significant differences in diabetes and obesity (diabetes: P = 0.375; obesity: P = 0.184).

**Table 1:** Comparison of demographic characteristics between two groups.

Parameters	Non-thrombosis group (n=244)	Thrombosis group (n=65)	t/χ^2^	P
Age (years)	39.52 ± 7.41	42.18 ± 6.36	2.648	0.009
Gender [n(%)]			2.287	0.130
Female	225 (92.21%)	56 (86.15%)		
Male	19 (7.79%)	9 (13.85%)		
BMI (kg/m^2^)	23.15 ± 3.07	23.83 ± 3.26	1.561	0.119
Smoking history [n(%)]			10.066	0.002
Yes	86 (35.25%)	37 (56.92%)		
No	158 (64.75%)	28 (43.08%)		
Drinking history [n(%)]			2.033	0.154
Yes	38 (15.57%)	15 (23.08%)		
No	206 (84.43%)	50 (76.92%)		
Cardiovascular risk factors				
Arterial hypertension [n(%)]	60 (24.59%)	27 (41.54%)	7.289	0.007
Hyperlipidemia [n(%)]	91 (37.30%)	30 (46.15%)	1.691	0.194
Diabetes mellitus [n(%)]	41 (16.8%)	14 (21.54%)	0.787	0.375
Obesity [n(%)]	59 (24.18%)	21 (32.31%)	1.767	0.184

BMI, body mass index.

In the comparison of disease characteristics, there was no significant difference in disease duration between the two groups (P = 0.220) (**Table 2**). The systemic lupus erythematosus disease activity index (SLEDAI) score was significantly higher in the thrombosis group compared to the non-thrombosis group (P<0.001), indicating higher SLE activity in the thrombosis group. There were no significant differences in the incidence of LN and APS between the two groups (all P>0.05). The proportion of patients using antimalarial drugs was not significantly different between the two groups (P = 0.428), but the proportion of patients using DMARDs was significantly higher in the thrombosis group compared to the non-thrombosis group (P = 0.009), suggesting that patients in the thrombosis group more frequently used DMARDs for treatment.

**Table 2:** Comparison of disease features between two groups.

Parameters	Non-thrombosis group (n=244)	Thrombosis group (n=65)	t/χ^2^	P
Disease duration (years)	6.65 ± 1.82	6.97 ± 2.18	1.229	0.220
SLEDAI score (points)	12.45 ± 1.62	14.29 ± 2.88	4.945	< 0.001
LN [n(%)]			2.005	0.157
Yes	100 (40.98%)	33 (50.77%)		
No	144 (59.02%)	32 (49.23%)		
APS [n(%)]			1.628	0.202
Yes	106 (43.44%)	34 (52.31%)		
No	138 (56.56%)	31 (47.69%)		
Use of antimalarial [n(%)]			0.627	0.428
Yes	103 (42.21%)	31 (47.69%)		
No	141 (57.79%)	34 (52.31%)		
Use of DMARD [n(%)]			6.772	0.009
Yes	88 (36.07%)	35 (53.85%)		
No	156 (63.93%)	30 (46.15%)		

SLEDAI, systemic lupus erythematosus disease activity index; LN, lupus nephritis; APS, antiphospholipid syndrome; DMARD, disease-modifying anti-rheumatic drug.

### 3.2 Routine coagulation indicators

PT was higher in the thrombosis group compared to the non-thrombosis group (P = 0.002) (**Figure 1**). APTT was also higher in the thrombosis group compared to the non-thrombosis group (P = 0.004). There were no significant differences in TT and FIB levels between the two groups (TT: P = 0.429; FIB: P = 0.839).

**Figure 1:**
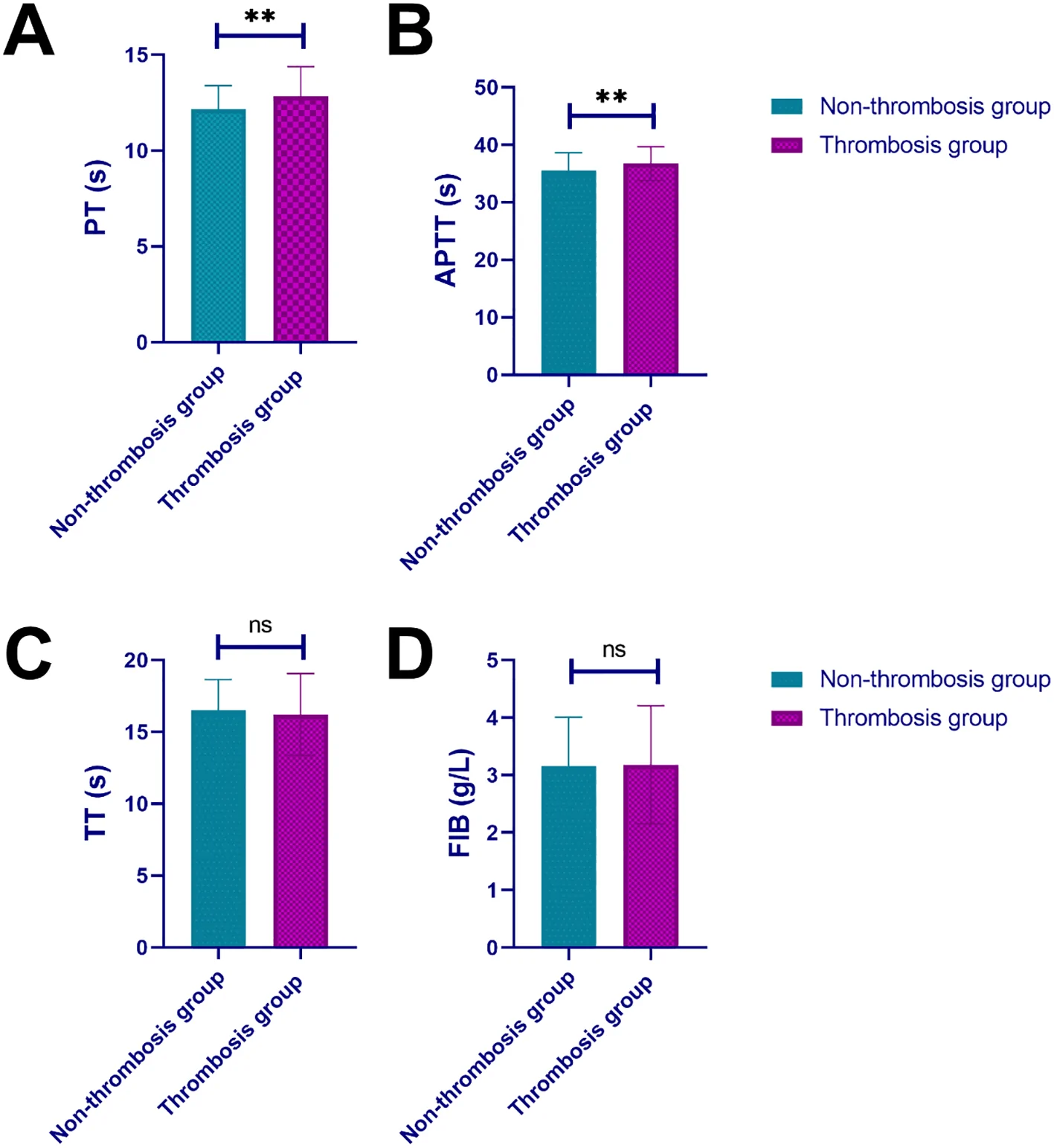
Comparison of routine coagulation indicators between two groups. **(A)** PT; **(B)** APTT; **(C)** TT; **(D)** FIB. PT, prothrombin time; APTT, activated partial thromboplastin time; TT, thrombin time; FIB, fibrinogen. ns, no significance; **P<0.01.

To investigate the potential contribution of LA positivity to APTT prolongation, we performed a post-hoc subgroup analysis. Patients with LA positivity had significantly longer APTT than LA-negative patients (P < 0.001), indicating that the observed APTT prolongation in the thrombosis group is largely attributable to the higher prevalence of LA in that group. PT prolongation may reflect the presence of LA in some assays, concurrent warfarin use, or other unmeasured confounders. Therefore, these routine coagulation parameters should be interpreted with caution and do not necessarily indicate a genuine hypercoagulable state.

### 3.3 aPLs positivity rates

Comparison of the aPLs positivity rates between the two groups showed that the proportion of aCL IgG/IgM positivity was higher in the thrombosis group compared to the non-thrombosis group (P = 0.001) (**Table 3**). The proportion of a-β2GPI IgG/IgM positivity was also higher in the thrombosis group compared to the non-thrombosis group (P = 0.003). The proportion of LA positivity was similarly higher in the thrombosis group compared to the non-thrombosis group (P = 0.002). Patients in the thrombosis group exhibited a higher rate of aPLs positivity.

**Table 3:** Comparison of aPLs positivity rates between two groups [n(%)].

Parameters	Non-thrombosis group (n=244)	Thrombosis group (n=65)	χ^2^	P
aCL IgG/IgM positive	56 (22.95%)	28 (43.08%)	10.503	0.001
a-β2GPI IgG/IgM positive	48 (19.67%)	24 (36.92%)	8.547	0.003
LA positive	15 (6.15%)	12 (18.46%)	9.760	0.002

aPLs, antiphospholipid antibodies; aCL, anticardiolipin antibody; a-β2GPI, anti-β2 glycoprotein I; LA, lupus anticoagulant; IgG, immunoglobulin G; IgM, immunoglobulin M.

### 3.4 LA ratio and aGAPSS

Both LA ratio and aGAPSS score were significantly higher in the thrombosis group than in the non-thrombosis group (LA ratio: P<0.001; aGAPSS: P<0.001), indicating that patients who developed thrombotic events had greater serological prothrombotic activity and a higher cumulative risk burden (**Figure 2**). These findings underscore the discriminative value of LA ratio and aGAPSS for identifying SLE patients at elevated thrombotic risk and support their integration into the combined predictive model.

**Figure 2:**
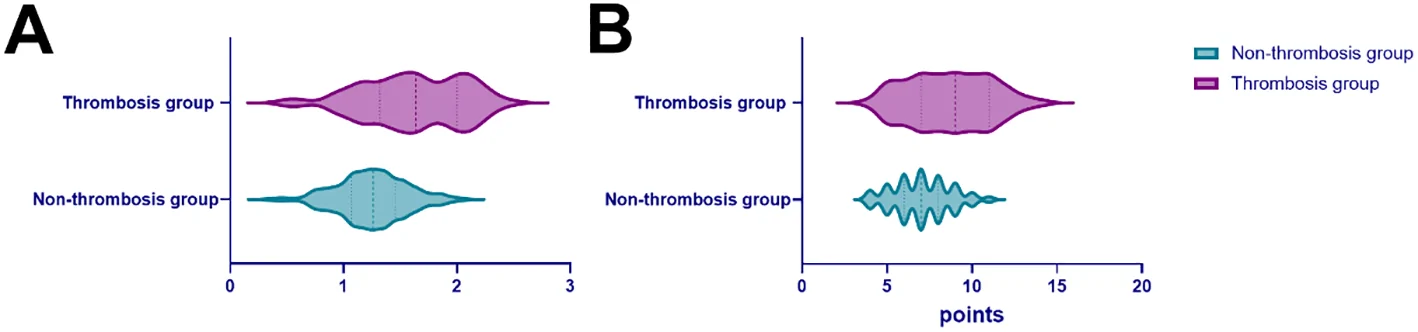
Comparison of LA ratio and aGAPSS between two groups. **(A)** LA ratio; **(B)** aGAPSS. LA, lupus anticoagulant; aGAPSS, adjusted global antiphospholipid syndrome score.

### 3.5 Logistic regression

The variable selection process was used LASSO regression (**Figure 3**). Panel A displays the coefficient shrinkage paths for all candidate predictors as the penalty parameter λ increases; each curve represents a variable’s coefficient trajectory (**Figure 3A**). Panel B presents the 10-fold cross-validation curve with binomial deviance plotted against log(λ); the vertical dashed lines mark the optimal λ values—the minimum error (λ.min) and the largest λ within one standard error of the minimum (λ.1se) (**Figure 3B**). The λ.1se was chosen to balance model fit and parsimony. This procedure identified five predictors with non-zero coefficients: age, smoking history, SLEDAI score, LA ratio, and aGAPSS. These variables were subsequently included as independent risk factors in the multivariate logistic regression model for predicting in-hospital thrombosis in SLE patients.

**Figure 3:**
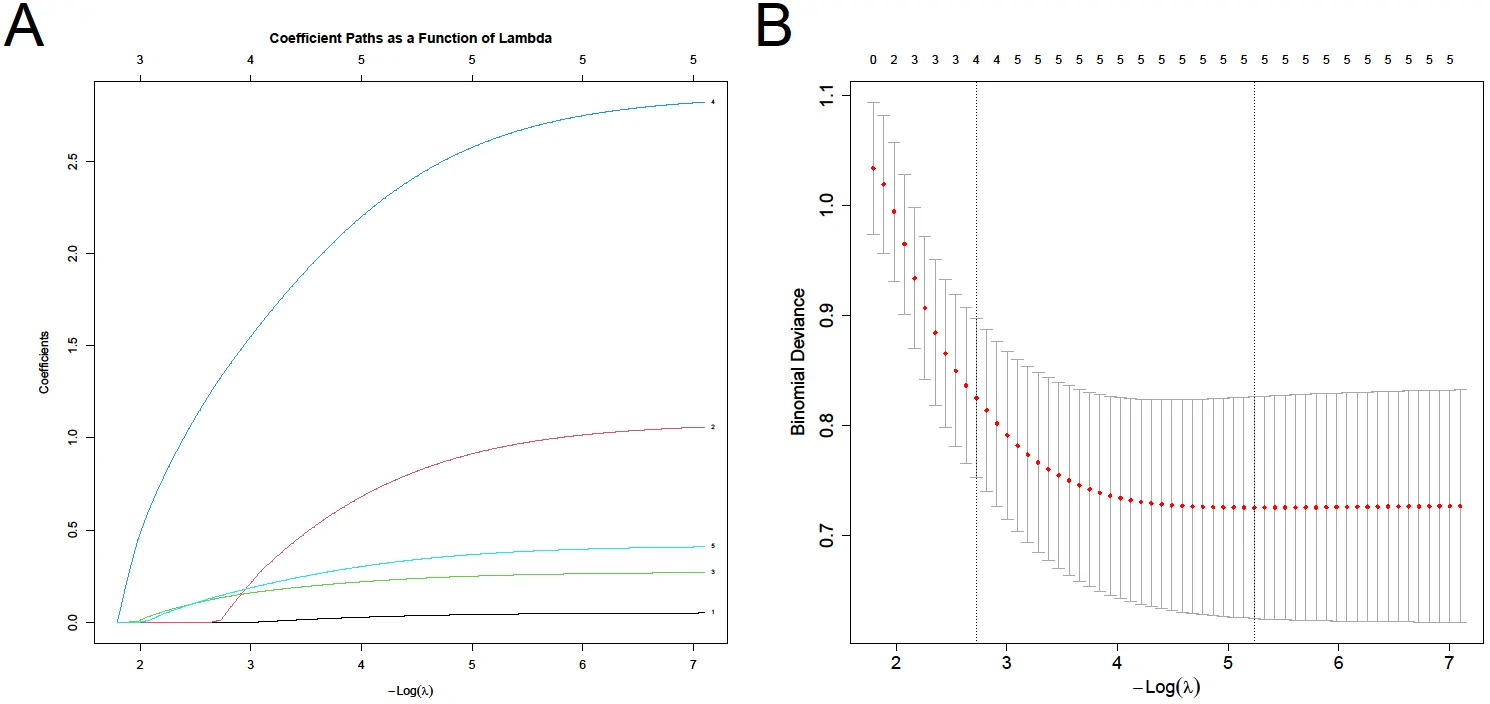
Risk factors selection via LASSO regression **(A)** Coefficient path as a function of lambda; **(B)** 10-fold cross validation. LASSO, least absolute shrinkage and selection operator.

In the multivariate logistic regression analysis, age (P = 0.0,48 odds ratio [OR]=1.053), history of smoking (P = 0.003, OR = 2.945), SLEDAI score (P = 0.001, OR = 1.312), LA ratio (P<0.011, OR = 17.432), and aGAPSS (P<0.001, OR = 1.514) all showed significant associations as risk factors, with OR greater than 1, indicating they increase the risk of thrombosis (**Table 4**).

**Table 4:** Multivariate logistic regression analysis of risk factors for thrombosis in SLE.

Parameters	Coefficient	Std error	Wald stat	P	OR	OR CI lower	OR CI upper
Age	0.051	0.026	1.980	0.048	1.053	1.001	1.107
Smoking history	1.080	0.364	2.967	0.003	2.945	1.443	6.013
SLEDAI score	0.271	0.084	3.245	0.001	1.312	1.113	1.545
LA ratio	2.858	0.547	5.229	<0.001	17.432	5.971	50.892
aGAPSS	0.415	0.098	4.249	<0.001	1.514	1.251	1.834

OR, odds ratio; CI, confidence interval; SLEDAI, systemic lupus erythematosus disease activity index; LA, lupus anticoagulant; aGAPSS, adjusted global antiphospholipid syndrome score.

### 3.6 ROC analysis

In the ROC analysis of the individual predictive performance of each risk factor for in-hospital thrombosis, the LA ratio yielded the highest AUC (0.751), followed by SLEDAI score (AUC = 0.710) and aGAPSS (AUC = 0.693) (**Table 5**). At the optimal cutoff of 1.545, the LA ratio demonstrated a sensitivity of 0.585 and specificity of 0.844, with a Youden index of 0.429 and F1 score of 0.539. The SLEDAI score (cutoff = 14.240) showed moderate sensitivity (0.523) but high specificity (0.869), whereas aGAPSS (cutoff=9.300) exhibited the highest specificity (0.906) albeit lower sensitivity (0.462). Age and smoking history had relatively modest AUCs (0.610 and 0.608, respectively). These findings suggest that LA ratio and SLEDAI score are the strongest individual discriminators, while aGAPSS offers excellent rule-out capability for excluding low-risk patients.

**Table 5:** ROC analysis of risk factors for thrombosis in SLE.

Parameters	Best threshold	Sensitivities	Specificities	AUC	Youden index	F1 score
Age	40.840	0.646	0.570	0.610	0.216	0.396
Smoking history	0.500	0.569	0.648	0.608	0.217	0.394
SLEDAI score	14.240	0.523	0.869	0.710	0.392	0.519
LA ratio	1.545	0.585	0.844	0.751	0.429	0.539
aGAPSS	9.300	0.462	0.906	0.693	0.368	0.508

AUC, area under the curve; SLEDAI, systemic lupus erythematosus disease activity index; LA, lupus anticoagulant; aGAPSS, adjusted global antiphospholipid syndrome score.

### 3.7 Combined model

Given that the aGAPSS is a validated instrument that includes LA as a binary component, we constructed the combined model by adding clinical covariates (age, smoking history, SLEDAI score) to the aGAPSS (**Table 6**). The combined model (AUC 0.832) significantly outperformed the aGAPSS alone (ΔAUC 0.139, P<0.001). The NRI of 0.356 indicates that 35.6% of patients were correctly reclassified into higher or lower risk categories compared to aGAPSS alone, while the IDI of 0.142 reflects a substantial improvement in overall discrimination. These findings demonstrate that clinical factors provide incremental predictive value beyond the original aGAPSS.

**Table 6:** Comparison of predictive performance between aGAPSS-alone and combined model.

Model	AUC (95% CI)	ΔAUC (95% CI)	P	NRI (95% CI)	IDI (95% CI)
aGAPSS alone	0.693 (0.643-0.743)	–	–	–	–
Combined model (aGAPSS + clinical [age + smoking history + SLEDAI score])	0.832 (0.787–0.877)	0.139 (0.095–0.183)	<0.001	0.356 (0.241-0.471)	0.142 (0.098-0.186)

AUC, area under the curve; CI, confidence interval; SLEDAI, systemic lupus erythematosus disease activity index; aGAPSS, adjusted global antiphospholipid syndrome score; NRI, net reclassification improvement; IDI, integrated discrimination improvement.

**Figure 4** illustrates the performance of the combined predictive model. Bootstrap internal validation showed good calibration, with an optimism-corrected slope of 0.935, indicating close agreement between predicted and observed thrombosis probabilities (**Figure 4B**). DCA revealed that the model provided positive net benefit across a wide range of threshold probabilities, supporting its clinical utility for identifying high-risk hospitalized SLE patients and guiding prophylactic anticoagulation decisions (**Figure 4C**).

**Figure 4:**
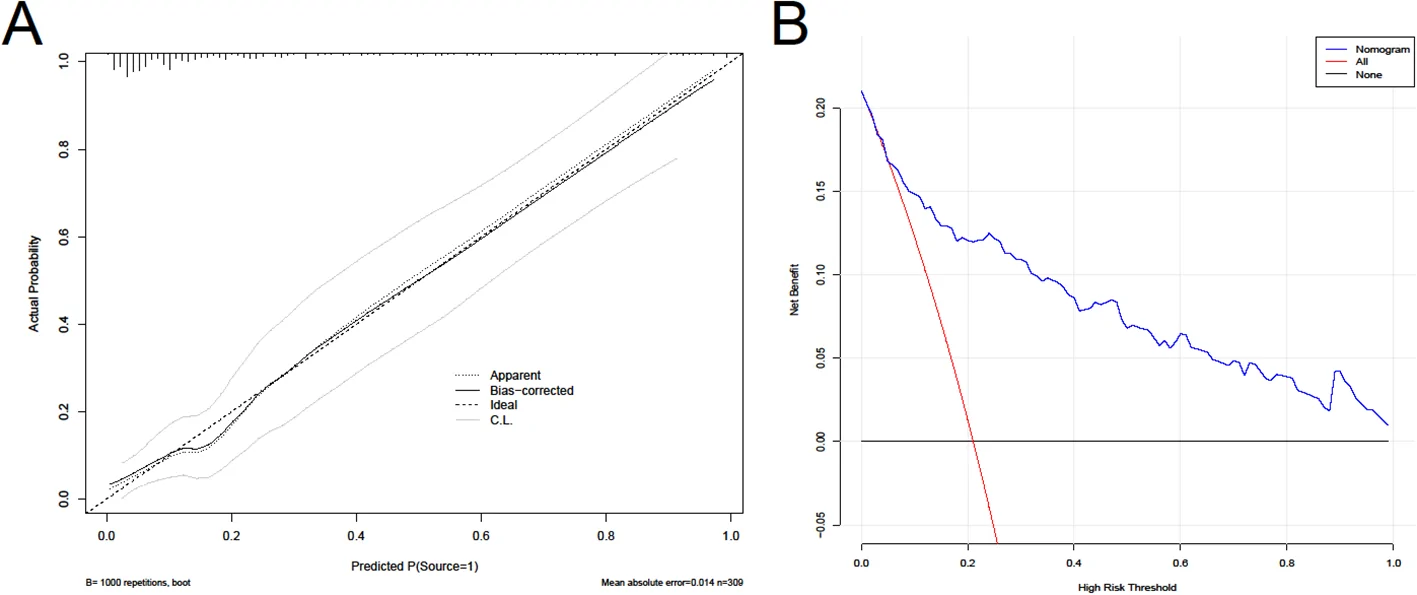
Performance analysis of the combined model. **(A)** Calibration plot; **(B)** Decision curve.

## 4 Discussion

This study systematically evaluated the predictive value of aPLs combined with aGAPSS for in-hospital thrombosis in a cohort of 309 hospitalized SLE patients. Our findings demonstrate that a comprehensive model integrating age, smoking history, SLEDAI score, and aGAPSS achieved good discriminative ability (AUC = 0.832), significantly outperforming aGAPSS alone (ΔAUC = 0.139, *P* < 0.001). These results provide a quantifiable, multifactorial risk assessment tool for in-hospital​ thrombosis risk stratification in hospitalized SLE patients. Before informing routine clinical practice, external validation and prospective studies are needed.

Among demographic and clinical variables, age emerged as an independent predictor, consistent with the known age-related increase in thrombotic risk in SLE populations (Płazak and Drabik, 2023). Smoking history was also independently associated, corroborating findings from large cohort studies that identify smoking as a modifiable risk factor for atherosclerosis and thrombosis (Verstovsek et al., 2023; Ishida et al., 2024). Hypertension, although not retained in the final LASSO-selected model, has been consistently linked to accelerated atherosclerosis and endothelial dysfunction in SLE (Dai et al., 2025; Mota et al., 2025). Notably, hyperlipidemia was not independently significant, possibly because its contribution is partially captured by the aGAPSS, which assigns 3 points for hyperlipidemia.

Disease activity, measured by the SLEDAI score, was a robust independent predictor. This finding supports the concept that active inflammation promotes a hypercoagulable state through cytokine release, neutrophil extracellular traps, and complement activation (Reshetnyak and Nurbaeva, 2023; Yao et al., 2023). A recent meta-analysis of 25 studies (13,290 patients) confirmed that high SLEDAI scores are associated with increased thrombotic risk (OR = 1.11, 95% CI: 1.05–1.16) (Shui et al., 2026). In our cohort, SLEDAI score demonstrated an AUC of 0.710 with high specificity (0.869), underscoring its utility in identifying patients at lower risk of in-hospital thrombosis. Notably, both groups demonstrated high disease activity (SLEDAI > 10), reflecting the severity of illness in this hospitalized cohort. Although the absolute difference in mean SLEDAI scores between groups was 1.84 points, this difference is clinically meaningful for several reasons. First, in our multivariate model, each 1-point increase in SLEDAI was associated with a 31.2% increase in thrombosis odds, indicating that even modest increments in disease activity confer substantial risk. Second, the minimal clinically important difference for SLEDAI has been reported as 3–4 points, and our observed difference approaches this threshold at the group level, with considerable individual variability extending well beyond. Third, the finding that both groups have high SLEDAI scores underscores that hospitalized SLE patients—regardless of thrombosis status—represent a population with significant disease activity requiring close monitoring. The key clinical message is that higher disease activity within this already-high-risk population further elevates thrombotic risk in a dose-dependent manner.

LA ratio showed the strongest association with thrombosis among individual parameters, consistent with the established hierarchy of aPL pathogenicity where LA confers the highest thrombotic risk (Raschi et al., 2024; Yang et al., 2025). LA ratio also demonstrated the highest individual discriminative ability among all assessed parameters, with excellent specificity. This superior performance likely reflects LA’s direct interference with the coagulation cascade and its ability to promote a prothrombotic state through multiple mechanisms, including endothelial activation, platelet aggregation, and complement pathway activation (Zhang et al., 2023). However, because LA is already incorporated as a binary component within the aGAPSS, we did not include the continuous LA ratio in the combined model to avoid redundancy. The continuous LA ratio remains a valuable independent predictor in the multivariate analysis, and its strong association with thrombosis underscores the importance of LA testing in clinical practice.

Importantly, aGAPSS remained an independent predictor after adjusting for other parameters, indicating that the score captures additional risk from the simultaneous presence of multiple aPL subtypes and from non-aPL components (hypertension, hyperlipidemia). This supports the clinical utility of aGAPSS as an integrated tool rather than a mere surrogate for individual antibody testing (Radin et al., 2019). Recent studies have validated aGAPSS in various populations, including Chinese SLE patients. Yu et al. demonstrated that aGAPSS of 3.5 or more had the best diagnostic accuracy for predicting aPL-related thrombosis in Chinese SLE patients (Yu et al., 2024). Furthermore, Karandyszowska et al. reported that for each point of aGAPSS, the hazard ratio for recurrent thrombosis increased by 10% (HR = 1.10, 95% CI: 1.01–1.19), corroborating the dose-response relationship observed in our study (Karandyszowska et al., 2025).

Our comparison of nested models demonstrated significant incremental value of the combined approach. Adding clinical variables (age, smoking, SLEDAI) to aGAPSS increased AUC from 0.693 to 0.832. The NRI of 0.356 indicates that 35.6% of patients were correctly reclassified into higher or lower risk categories compared to aGAPSS alone. This finding aligns with emerging evidence that combining aGAPSS with routine laboratory parameters improves predictive performance (Yu et al., 2024). The IDI of 0.142 reflects a substantial improvement in overall discrimination, suggesting that relying solely on aGAPSS would miss substantial thrombotic risk captured by clinical factors. Therefore, a comprehensive assessment integrating both the validated aGAPSS and clinical domains is warranted for optimal risk stratification.

Several limitations should be considered. First, this single-center retrospective study may limit generalizability, and the lack of an external validation cohort means our optimism-corrected performance metrics require confirmation in independent datasets. Second, the information on anticoagulation therapy—including the type, dose, indication, and timing of administration—was not systematically captured in our retrospective dataset. This is an important limitation because anticoagulation directly influences thrombotic event rates and may confound the association between baseline risk factors and incident thrombosis. While patients who developed in-hospital thrombosis may have received anticoagulation only after the event, which would not affect baseline predictive variables, this does complicate the interpretation of time-dependent relationships. Future prospective studies should collect detailed longitudinal anticoagulation data to allow for time-updated covariate adjustment and more robust causal inference. Third, follow-up was restricted to hospitalization, so we could not assess long-term thrombotic outcomes or recurrent events. Fourth, we did not distinguish arterial from venous thrombosis, despite evidence that these entities have distinct risk factor profiles—venous thrombosis is strongly driven by aPLs, while arterial events are predominantly associated with traditional cardiovascular risk factors. Fifth, our findings apply only to adult SLE patients; pediatric populations may differ. Sixth, aPL measurements were obtained at a single time point at admission, without repeat testing at 12 weeks apart as recommended by the 2023 ACR/EULAR APS classification criteria. This reflects the pragmatic nature of acute hospital-based risk prediction, where clinical decisions regarding prophylactic anticoagulation must be made based on immediately available laboratory data. Our primary objective was short-term prediction of in-hospital thrombosis, and the aGAPSS has been validated in prior studies using single time-point measurements. However, we acknowledge that single time-point testing does not distinguish persistent from transient aPL positivity, which may affect long-term risk stratification. Seventh, the variable “APS” in our analysis relied on documented clinical diagnoses from medical records rather than systematic application of classification criteria by the study team. This may have led to under-ascertainment of APS in patients who had both thrombosis and aPL positivity but were coded under specific event diagnoses or lacked 12-week confirmatory testing. Future studies should apply classification criteria uniformly to determine the true prevalence of APS in SLE cohorts. Finally, as this study was limited to in-hospital thrombosis in a single-center retrospective cohort, the generalizability of the model to outpatients, stable SLE populations, or long-term thrombotic risk prediction remains unestablished. The model should therefore be regarded as a proof-of-concept​ for in-hospital risk stratification rather than a validated clinical decision tool.

## 5 Conclusion

This study demonstrates that a comprehensive model incorporating aGAPSS with clinical covariates (age, smoking history, SLEDAI score) provides good discriminative performance for in-hospital thrombosis among hospitalized SLE patients, outperforming aGAPSS alone. While these findings support the model’s potential for risk stratification, its current role is limited to research settings and hypothesis generation. Direct guidance of prophylactic anticoagulation requires prospective validation in independent cohorts.

## Data Availability

The raw data supporting the conclusions of this article will be made available by the authors, without undue reservation.
